# Colloidal density control with Bessel–Gauss beams

**DOI:** 10.1038/s41598-021-91638-w

**Published:** 2021-06-10

**Authors:** Cristian Hernando Acevedo, Ruitao Wu, J. Keith Miller, Eric G. Johnson, Aristide Dogariu

**Affiliations:** 1 CREOL, The College of Optics and Photonics, University of Central Florida, Orlando, FL USA; 2 COMSET, Holcombe Department of Electrical and Computer Engineering, Clemson University, Clemson, SC 29634 USA

**Keywords:** Materials science, Optics and photonics

## Abstract

Optical manipulation of colloidal systems is of high interest for both fundamental studies and practical applications. It has been shown that optically induced thermophoresis and nonlinear interactions can significantly affect the properties of dense colloidal media. However, macroscopic scale phenomena can also be generated at thermal equilibrium. Here, we demonstrate that steady-state variations of particle density can be created over large, three-dimensional regions by appropriately structured external optical fields. We prove analytically and experimentally that an optical vortex beam can dynamically control the spatial density of microscopic particles along the direction of its propagation. We show that these artificial steady-states can be generated at will and can be maintained indefinitely, which can be beneficial for applications such as path clearing and mass transportation.

## Introduction

Heterogeneous systems of insoluble elements dispersed throughout a continuous medium are ubiquitous realizations of physical systems. Colloidal systems comprising particulates of varying sizes, from atomic to macroscopic scales, have unique spatial and temporal characteristics that are central to phenomena in chemistry, biology, environmental science, materials science, etc. Dynamic control of their properties is critical for a variety of applications.

Mesoscopic colloidal systems are used as working models for condensed matter for several reasons^1^. Most importantly, the characteristic time and spatial scales are easily accessible experimentally. Moreover, the effective interaction between colloidal particles can be conveniently tuned by modifying their intrinsic properties or by applying suitable external fields. Thus, one can conveniently study phenomena that are otherwise difficult or practically impossible to probe in atomic systems^2,3^ and in specific realizations of active matter^4,5^.

Colloidal dynamics is driven in general by a combination of intrinsic, thermally activated motion and an external advective influence due to external fields. Aside from the trivial example of gravity, advective transport at large Péclet numbers can be realized by applying external fields such as electric^6,7^, magnetic^8–10^, thermal^11–13^, chemical^14,15^, osmotic^16,17^, or electromagnetic^18^. For a number of experimental reasons, optical fields are particularly appealing. Most notable is the ability to suitably control the external influence by adjusting properties such as intensity, polarization, coherence, etc. Starting from the earlier demonstrations by Ashkin^19,20^, structured light beams were successfully used to transfer linear momentum and create forces of the order of piconewtons^21^ or angular momenta and induce deterministic rotations of suspended particulates^22–28^. Nowadays, light is routinely used to control, move and accelerate, individual nano and microscopic objects in either two- and three-dimensional environments medium^29–33^.

Physical properties of colloidal systems can also be regulated at macroscopic scales^34–36^. Moreover, these reports focus on manipulating the overall properties of lower dimensionality^37^ or macroscopically homogeneous media^38^. A dynamic alteration of the optical transmittance through a colloidal medium has also been proposed^39^.

At high densities of particulates, colloidal systems can sometimes be regarded as effective media through which waves propagate. Optical action, together with interactions driven by thermophoretic and hydrodynamic forces, can even lead to nonlinear regimes of propagation and behave as, for instance, artificial Kerr media^40–42^, or Raman-active media^43^.

Here we demonstrate experimentally that large scale inhomogeneities can be created and maintained for a long time by applying relatively low-power electromagnetic fields. The density of suspended particulates can vary spatially and transitions between different steady-state conditions can be easily followed by varying the properties of the external fields. We present an analytical model that describes quantitatively how the electromagnetic force exerted by an optical vortex beam controls the local density and flux of Brownian particles in a three-dimensional colloidal medium.

## Results

### Particle density in colloidal systems

Colloidal systems are subject to both thermally driven internal fluctuations and external forces. When the applied forces vary slowly in time compared with all other relevant time scales, a steady state can be reached where the concentration of particles is spatially non-uniform and the particle-associated fluxes are constant. At equilibrium, in a large-scale medium when the effect of boundaries and the finite amount of particles can be neglected, creating local variations of concentration requires external force fields that are spatially structured.

Microscopically, an individual particle in a colloidal suspension exposed to an external force can be effectively considered as executing a diffusing motion that is biased along the direction of the external force. At macroscopic scales, when one examines the collective motion of many of such particles, it is often convenient to describe the system in terms of a locally averaged number density $\rho \left( {{\varvec{r}},t} \right)$ of particles and assign a local velocity field ${\varvec{u}}\left( {{\varvec{r}},t} \right)$ that represents the average over much faster individual velocities ${\varvec{v}}\left( {{\varvec{r}},t} \right)$. Thus, the internal dynamics of the colloidal system can be described in terms of a directional flux that points along the direction of the local velocity field ${\varvec{u}}\left( {{\varvec{r}},t} \right)$. Based on the continuity equation, the time-varying density around a particular location ${\varvec{r}}$ is given by (See Section 1 in Supplementary Materials)

1 $$ \rho \left( {{\varvec{r}},t} \right) = \rho_{0} e^{{ - \mathop \smallint \limits_{0}^{t} \nabla \cdot{\varvec{u}}\left( {{\varvec{r}},t^{\prime}} \right) dt^{\prime}}} $$

where $\rho_{0}$ denotes the initially uniform density of particles. In the absence of an external force field, the local velocity ${\varvec{u}}\left( {{\varvec{r}},t} \right)$ is determined by the ensemble average over randomly oriented thermal forces and, therefore, the exponent in Eq. (1) averages to zero. An external force field, however, can modify the local density both spatially and temporally.

### Density variations due to an optical vortex beam

**Figure 1:**
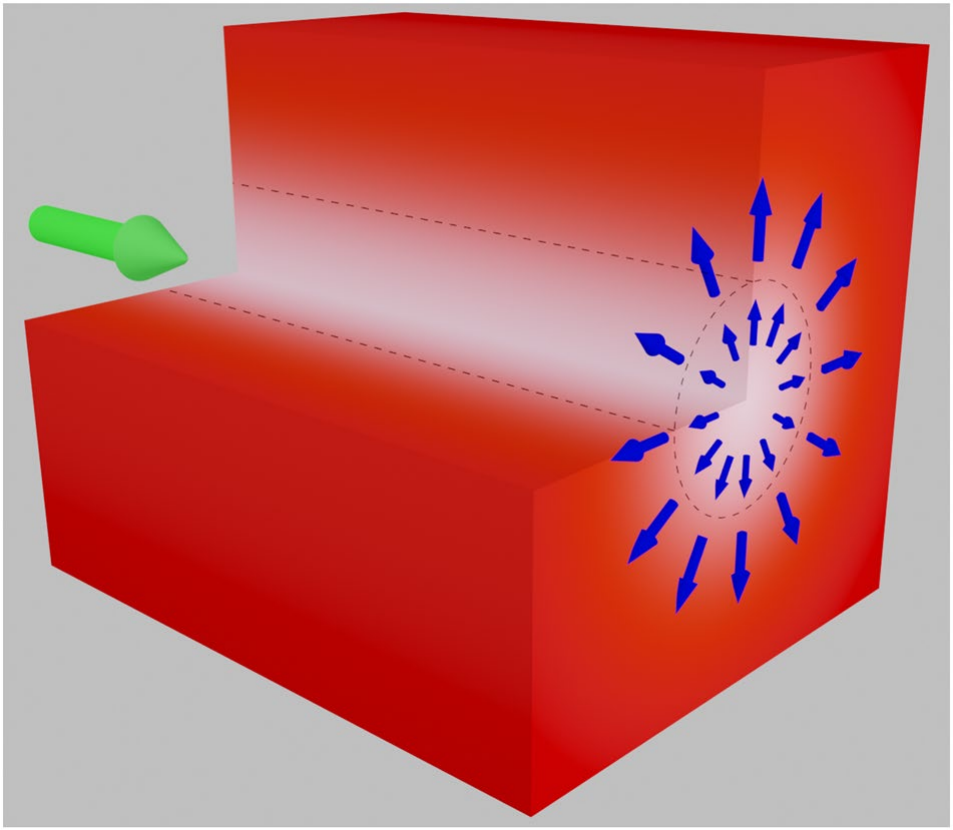
Transversal distribution of force exerted by a vortex beam on a generic colloidal system. The force field generates a flux of particles across the surface of a cylindrical volume oriented along the propagation direction of the beam.

Let us examine the particular situation where a particle flux is created through a cylindrical surface as illustrated in Fig. 1. With respect to this surface, the particles can be either *inside* or *outside* the cylinder. In addition, let us assume that the external forces act along the radial direction but only in the proximity of this surface. In these conditions, it is possible to evaluate the final particle density in the colloidal system. If the force field is azimuthally symmetric, the problem can be fully described as a one-dimensional process.

An external force acting locally can be easily generated electromagnetically, for instance, by a structured field such as an optical vortex beam. Rigorously, the force field comprises both conservative and non-conservative components^44^, and the particles are subject to longitudinal and transversal forces^45^. We will first analyze the effect of the transversal forces, which are dominated by intensity gradients^29^.

For particles much smaller than the wavelength, the time averaged electromagnetic force depends on intensity $I$*,* Poynting vector ${\varvec{S}}$, and spin flux ${\varvec{L}}_{{\varvec{s}}}$ ^45^. The time average is sought over many oscillation periods of the electromagnetic field. The force also depends on the scattering cross-section $\sigma$ of the particle and its polarizability $\alpha$. Let us consider that, at time $t = 0$, the colloidal system is exposed to a linearly polarized optical vortex field with topological charge $m$, beam waist $w,$ and a slowly varying envelope $A\left( t \right)$. Assuming further that the scattering is weak, one can show that the colloidal density varies like (See Section 1 in Supplementary Materials)

2 $$ \rho \left( {r,t} \right) = \rho_{0} e^{{ - f\left( r \right)\mathop \smallint \limits_{0}^{t} {\text{A}}\left( {t^{\prime}} \right) dt^{\prime}}} , $$

where the rate of change determined by $$f\left( r \right) = \frac{1}{r}\frac{dI}{{dt}}Re\left\{ \alpha \right\}\left[ { - \frac{{4r^{3} }}{{w^{4} }} - \frac{r}{{w^{2} }}\left( {4m + 2} \right) + \frac{{m^{2} }}{r}} \right].$$ If the field amplitude is constant, a steady-state establishes where the colloidal density acquires a radial profile $$\rho \left( {r,t} \right)$$ $$= \rho_{0} e^{ - f\left( r \right)t}$$ determined by the properties of the optical field. As long as the field is applied, at a radial position $$ r$$, the density continues to decreases exponentially with a rate determined by 1/$$ f\left( r \right).$$ Over the cross-section of the entire virtual cylinder of radius $$ R$$, the total density will vary as $${\Upsilon }\left( t \right) = \mathop \smallint \limits_{0}^{R} \rho \left( {r,t} \right)rdr.$$ The level of this density variation is controlled by the properties of the applied field. Of particular interest for practical applications is the variation of average density in response to changes in the intensity and the topological charge of the excitation beam as illustrated in Sections 2 and 3 of Supplementary Materials.

Once the external force field is turned off, the particles return to their initial state through free thermal motion. Therefore, the density of particles will evolves in time as $\rho \left( {r,t} \right) = \frac{{\rho \left( {r_{0} ,t_{0} } \right)}}{{\sqrt {2\pi D\left( {t - t_{0} } \right)} }}e^{{\frac{{\left( {r - r_{0} } \right)^{2} }}{{4D\left( {t - t_{0} } \right)}}}}$, at a rate determined solely by the thermal diffusion coefficient $D$.

### Experimental demonstration of particle density variation under vortex beam

**Figure 2:**
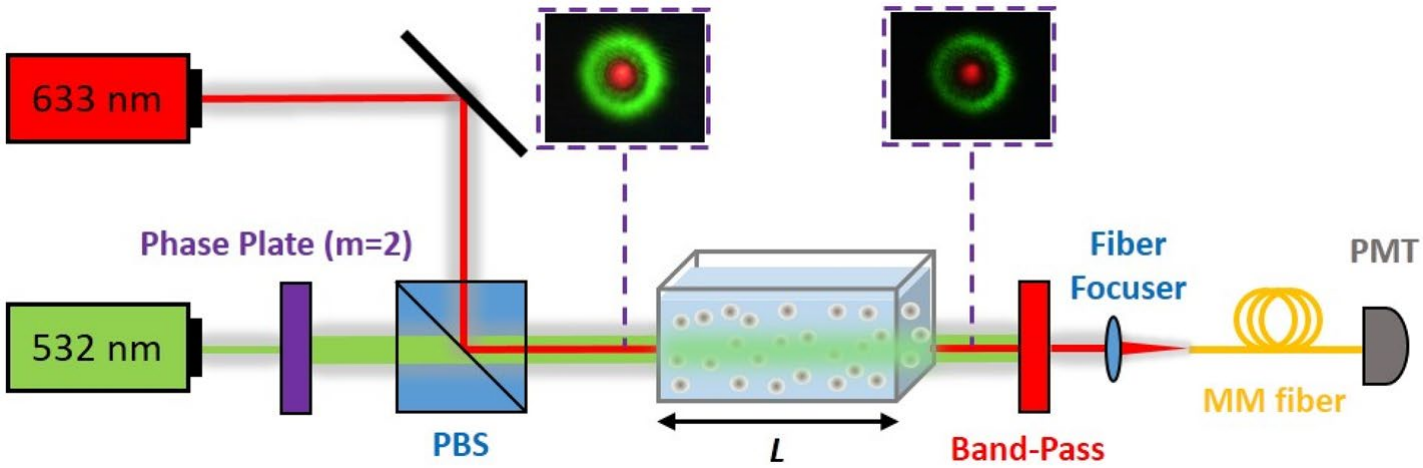
Schematic of experiment. An optical vortex beam with $m = 2$ is launched through a colloidal suspension. A coaxial Gaussian beam is used to continuously monitor the optical density.

We performed systematic experiments with small silica particles (22-nm in diameter) exposed to an optical vortex beam with topological charge value $m = 2$^46^. The longitudinal density variations were continuously monitored by a coaxially launched probe beam. The setup is illustrated in Fig. 2 and the details are included in the Method section.

**Figure 3:**
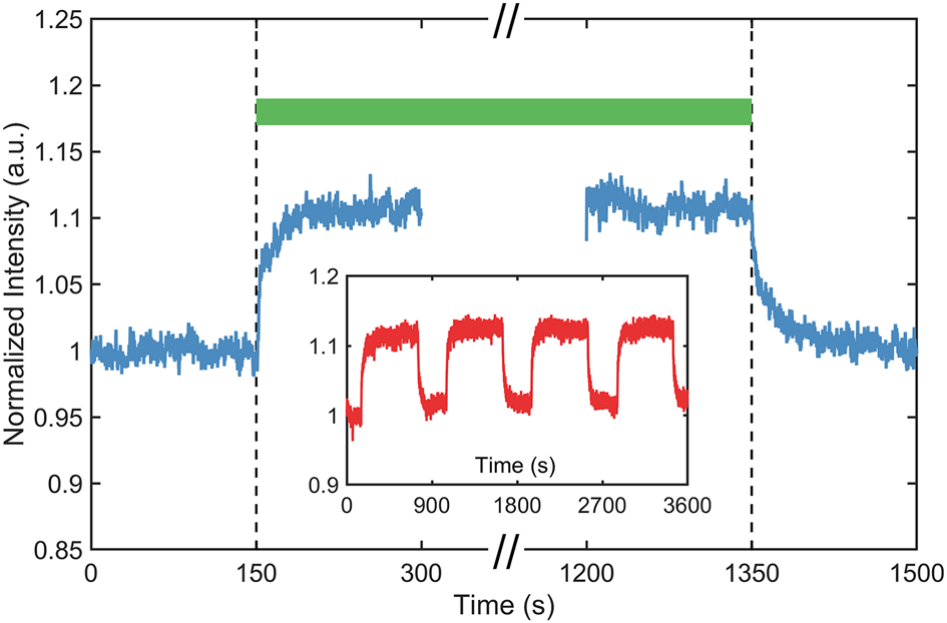
Normalized values of transmitted intensity during 1200 s of vortex field exposure. The green vortex field is off for the first and the last 150 s of each cycle. The inset shows the transmittance of the same sample during four exposure cycles.

A typical variation of intensity of the probe beam is illustrated in Fig. 3. As can be seen, during the initial 150 s, the colloid is at equilibrium and the optical density is constant in time. The very low power of the probe beam has a negligible effect on the particle density. The situation changes abruptly when the vortex beam is switched on. A radial force is now exerted on the colloidal particles and their density starts to change as described by Eq. (2); the optical density decreases and the transmission of the probe beam increases. After some time, a new steady-state is reached where the colloidal density is constant as long as the vortex beam is present. Once this field is switched off, the free diffusion of particles homogenizes the density gradients and the system evolves towards its state of initial equilibrium. The entire process can be repeated as shown in the inset.

**Figure 4:**
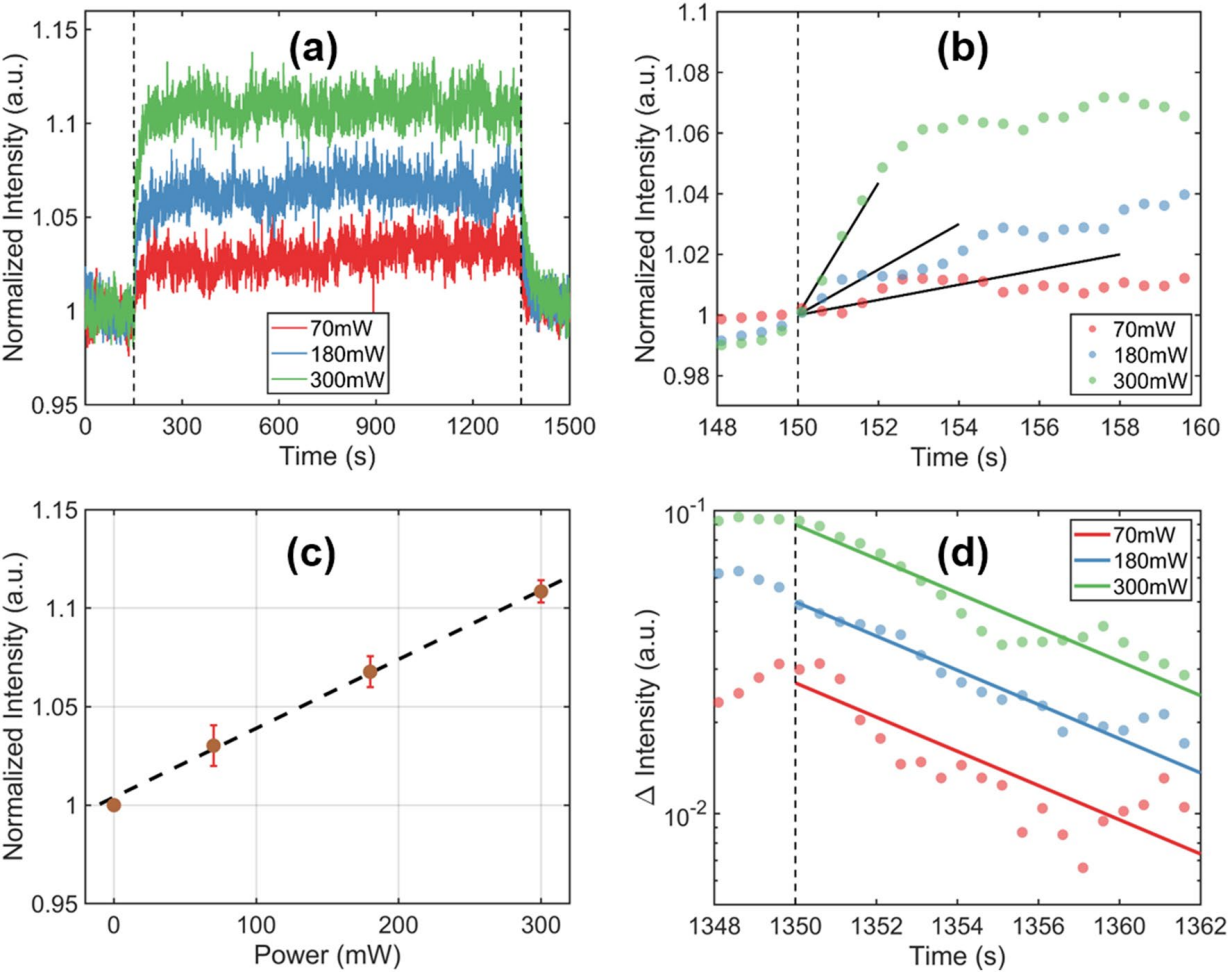
(**a**) Evolution of normalized transmittance for different power of the vortex beam as indicated. (**b**) Immediately after the field is applied, the transmitted intensity increases linearly in time (see text). (**c**) Normalized values of maximum transmitted intensity as function of applied power. (**d**) The rate of transmittance decrease after the external field is turned off at t = 1350 s.

We performed a systematic investigation of the particle density variation for different strengths of the external force field by controlling the power of the vortex beam. The results are summarized in Fig. 4 and a few general observations are in place. First, the role of power of the external field is evident in Fig. 4a; the stronger the applied field, the more significant the density modifications. Second, the transient regimes between the two steady states seem to share some similarities, which we will describe quantitatively in the following.

Let us first discuss the non-stationary transition after the external field is turned on. The time variation of the probe intensity is well described by the attenuation law $I\left( t \right) = I_{0} e^{ - L\sigma \rho \left( t \right)} ,$ where *L* is the propagation path, $\sigma$ is the scattering cross section of individual particle in the medium. Of course, the transmitted intensity remains constant as long as $\rho \left( t \right)$ does not change. After the external field is applied, the probe intensity $I$ increases at a rate that determines the density variation $\rho \left( t \right) = \mathop \smallint \limits_{0}^{{\Phi }} \rho \left( {r,t} \right)rdr$, which can be evaluated using Eq. (2) for a probe beam of size ${\Phi }$. It is straightforward to show that, in the limit $t \to 0$, i.e. at short times after the field is turned on, the rate of increase in transmittance follows $\frac{dI}{{dt}} \propto P.$ In other words, the initial rate depends on a geometric factor determined by the external field configuration and, importantly, it increases linearly with the applied power. This conclusion is verified in our experiments as indicated in Fig. 4b, where the initial rates of increase are shown to scale linearly with the power of the applied field.

As can be seen in Fig. 4c, a new steady-state emerges after a transition time starting around 600 s. In this phase, a balance is attained between the internal particle fluxes and, consequently, the density remains constant in time albeit non-uniform across the volume. For this steady state, $\frac{dI}{{dt}} = 0$, the maximum value of the detected intensity is proportional with the power of applied field: $I_{max} = I_{0} \propto P$. The error bars in Fig. 4c denote the standard deviation evaluated in different steady-state conditions.

Finally, once the external field is turned off, the system returns gradually to its initial condition. As mentioned before, in this transitory regime between two steady-states illustrated in Fig. 4d, the particle density evolves according to Fick’s law. The duration of this transition is determined solely by the intrinsic diffusion coefficient of the colloidal system and the spatially non-uniform density distribution imposed during the external excitation. The fact that the rate of transition to the initial state does not depend on the applied power indicates that (1) the diffusion coefficient is practically constant across the entire volume irrespective of local density of particles and (2) the boundary influences are negligible in our large-scale medium.

## Discussion and outlook

In summary, we demonstrated a convenient method for controlling the macroscopic properties of a colloidal systems. We have shown, theoretically and experimentally, that an optical vortex beam can be used to control the density of nanometric particles in a colloidal system. We have shown that an equilibrium situation can be maintained within a cylindrical region of depleted particle density, which is created as a result of transversal conservative forces exerted by the optical field.

The spatial extent of the induced density variations and the temporal scales of the transition between the two steady-states depend not only on the intrinsic thermodynamic and hydrodynamic properties of the colloid but also on the controllable properties of the applied optical field. This allows exploring the competition between Brownian dynamics and spatial confinement that leads to macroscopic phenomena such as three-dimensional giant breathing modes, which develop under time-dependent external fields.

We would also like to comment on the magnitude of the effect demonstrated here. First, it is evident that the depletion of the colloidal density depends on the magnitude of the external force, which can be efficiently enhanced by increasing the power of the optical field. However, another effect, apparent for crowded distributions of particles, could modify the effective particle flux. Long-range hydrodynamic interactions can actually amplify the effect the optical force ${\varvec{F}}$ acting on each particle and eventually lead to increase of its velocity ${\varvec{v}}_{i} = \sum \overline{\overline{{\varvec{\mu}}}}_{ij} {\varvec{F}}_{j}$ ^47^. This influence, whose magnitude is determined by an Oseen interaction tensor like $\overline{\overline{{\varvec{\mu}}}}_{ij} \propto \left( {\gamma R_{ij} } \right)^{ - 1} \left( {\overline{\overline{{\varvec{I}}}} + \hat{R}_{ij} \hat{R}_{ij} } \right),$ could significantly affect the time scales of the transition regions between the two steady-states.

The effect described here is not strictly limited by the size of colloidal particles. In fact, we observed similar trends in experiments comprising different particle sizes. Additional examples that illustrate the evolution of particle density and its dependence on the strength of the external field are included in the Supplementary Materials for micron size particles. In this case, however, an analytical description of the velocity fields is not available and the complex electromagnetic interaction has to be described numerically^48^.

Our demonstration concerned colloidal systems in thermal equilibrium. If the colloidal particles absorb light, additional phenomenology emerges^18^. Local thermal unbalance can lead to thermal gradients that affect the particles mobility and their ability to interact ^49,50^. Driven by thermophoresis, macroscopic convection currents can develop which can be adjusted by controlling the optical field. In such non-stationary conditions, the effects of an external field can be used not only to drive structural modifications in colloidal systems but also to conveniently mimic a range of other natural phenomena.

Finally, the present demonstration relying on field topology can be augmented by additional control of polarization and coherence of an optical beam. In the specific context of vortex beams, it would also be of interest to understand the macroscopic effects of phase gradient forces generated by fields with different topological charges. The full photonic control of particle density in macroscopic colloids may open up new means for guiding the structural evolution of systems where the interaction between parts is mediated by the electromagnetic field. This should be relevant to biological organizations (proteins and other cellular components), soft materials structuring (colloids and polymers), or emerging technologies for fabricating mechanical nanostructures (motors, ratchets, scaffolding).

## Methods

We performed systematic experiments using diluted suspensions of small silica particles (refractive index 1.46; diameter 22 nm) in distilled water. The colloidal system was placed in a cuvette (1 cm × 1.6 cm × 4 cm) and was illuminated by an optical vortex beam with topological charge value m = 2. The beam was generated by Gaussian laser beam with 2.5 mm in diameter (Coherent Genesis CX, wavelength 533 nm) passed through a suitable phase plate ^46^.

A secondary beam from a He–Ne laser of diameter 0.8 mm (Coherent, wavelength 633 nm, power 2.0 mW) was launched coaxially with the optical vortex beam and was used as “probe” beam for measuring the local variation of the particle density. The two beams were spatially overlapped using a polarizing beam splitter. When measuring the transmittance of the probe beam, a red-pass filter (Thorlabs, FELH0600) was used to isolate the scattering from the main vortex beam. The probe beam was collected with a multimode optical fiber and its intensity was detected using a photomultiplier (Hamamatsu, H6180).

## Supplementary Information

Supplementary Information.

## Data Availability

Data that supports the findings of this study are available from the corresponding author upon reasonable request.
