# Supplementary material for: Colloidal density control with Bessel–Gauss beams
Source: Sci Rep. 2021 Jun 10;11:12284. doi: 10.1038/s41598-021-91638-w (PMC8192777; doi:10.1038/s41598-021-91638-w)
Supplement: Supplementary file 1 — Supplementary Information. [file 41598_2021_91638_MOESM1_ESM.docx]

Supplementary materials for

Colloidal density control with Bessel-Gauss beams

Cristian Hernando Acevedo^1^, Ruitao Wu^1^, J. Keith Miller^2^, Eric G. Johnson^2^, and Aristide Dogariu^1*^

**^1^**CREOL, The College of Optics and Photonics, University of Central Florida, Orlando, Florida, USA.

^2^COMSET, Holcombe Department of Electrical and Computer Engineering, Clemson University, Clemson, SC 29634, USA.

Corresponding e-mail address: [adogariu@creol.ucf.edu](mailto:adogariu@creol.ucf.edu)

# Theoretical model

Here, we present a generic model of the particle density variation under external forces. Microscopically, the movement particle of mass $m$ is, in general, subject to random forces $\boldsymbol{\xi}\left( t \right)$ of thermal origin and possible external forces $\boldsymbol{F}_{ext}\left( t \right)$ ^1^. For times much longer than the ballistic time, $t\gg m/\gamma$, the instantaneous velocity is $\boldsymbol{v}\left( \boldsymbol{r},t \right)=\frac{1}{\gamma}\boldsymbol{F}_{ext}\left( \boldsymbol{r},t \right)+\frac{1}{\gamma}\boldsymbol{\xi}\left( t \right)$ with $\gamma$ being the inverse of particle’s mobility. The random force $\boldsymbol{\xi}\left( t \right)$ is a Wigner process of zero average $\left\langle\xi(t') \right\rangle=0$ and $\left\langle\xi(t')\xi(t'') \right\rangle=\frac{{2k}_{b}T}{\gamma}\delta\left( t'-t'' \right)$ at thermal equilibrium. In principle, the external force can vary in time and its evolution be characterized by $\left\langle\boldsymbol{F}_{ext}\left( \boldsymbol{r},t' \right)\boldsymbol{F}_{ext}\left( \boldsymbol{r},t'' \right) \right\rangle=F_{0}^{2}\left( \boldsymbol{r} \right)\Gamma\left( t'-t'' \right).$

Around a mean location $\boldsymbol{r}_{0}$, the one-dimensional displacement along the direction of the external field is $x\left( t \right)=x_{0}+\frac{1}{\gamma}\int_{0}^{t} \left[ \xi\left( t' \right)+F_{ext}\left( \boldsymbol{r}_{0},t' \right) \right]dt'$ and, if $\xi\left( t \right)$ and $F_{ext}(t)$ are uncorrelated, the corresponding mean square displacement is found to be

$\left\langle x^{2}(\boldsymbol{r}_{0},t) \right\rangle=Dt+\frac{F_{0}^{2}\left( \boldsymbol{r}_{0} \right)}{\gamma^{2}}\int_{0}^{t} \int_{0}^{t} \Gamma\left( t'-t'' \right)dt'dt''$. (S1)

where we used the definition of the thermal diffusion coefficient $D=\frac{2k_{B}T}{\gamma}$ . This means that, around any position $\boldsymbol{r}$, the particle executes a Brownian motion that is biased along the direction of the external force. The magnitude of this bias and its temporal variation are determined by the properties of the external force field.

In a colloidal system, a large amount of microscopic components are subject to similar Langevin equations leading to the diffusive motion as described in Eq. (S1). At macroscopic scales, when one examines the collective motion of many of such particles, it is often convenient to describe the system in terms of a locally averaged number density $\rho(\boldsymbol{r},t)$ of particles and assign a local velocity field $\boldsymbol{u}\left( \boldsymbol{r},t \right)$ that represents, at point $\boldsymbol{r}$, the average over much faster individual velocities $\boldsymbol{v}\left( \boldsymbol{r},t \right)$. Even though it is an average over many microscopic components, $\boldsymbol{u}\left( \boldsymbol{r},t \right)$ may vary spatially from point to point and also temporally, but at much larger time scales. At macroscopic scales, the internal dynamics of the colloidal system can be described in terms of a directional flux $\boldsymbol{J}\left( \boldsymbol{r},t \right)=\rho\left( \boldsymbol{r},t \right)\boldsymbol{u}\left( \boldsymbol{r},t \right)$ that points along the direction of the local velocity field $\boldsymbol{u}\left( \boldsymbol{r},t \right)$. Of course, when the diffusion of individual particles is unbiased, i.e. when external field in Eq. (S1) is absent, the average flux vanishes.

When there are no sources or sinks for the colloidal particles, the local density can only increase or decrease due to internal flows, say across a virtual surface$S$ within the medium. With respect to such a surface, the continuity equation requires $\frac{\partial\rho(\boldsymbol{r},t)}{\partial t}+\nabla\cdot\left[ \rho\left( \boldsymbol{r},t \right)\boldsymbol{u}\left( \boldsymbol{r},t \right) \right]=0$and, taking into account that$\dot{\rho}(\boldsymbol{r},t)= \frac{\partial\rho(\boldsymbol{r},t)}{\partial t}+\boldsymbol{u}\left( \boldsymbol{r},t \right)\cdot\nabla\rho(\boldsymbol{r},t)$ ^2^, it follows that$\dot{\rho}\left( \boldsymbol{r},t \right)+\rho\left( \boldsymbol{r},t \right)[\nabla\cdot\boldsymbol{u}\left( \boldsymbol{r},t \right)]=0$. The formal solution for the time-varying density around a particular location $r$ is

$\rho\left( \boldsymbol{r},t \right)={\rho_{0}e}^{-\int_{0}^{t} \nabla\cdot\boldsymbol{u}\left( \boldsymbol{r},t^{'} \right)dt^{'}}$. (S2)

where $\rho_{0}$ denotes the initial density of particles. The Eq. (S2) describes the spatiotemporal variation of the local density of particles that are individually executing a biased Brownian motion with an average mean square displacement given in Eq. (S1). In the absence of an external force field, the local velocity$\boldsymbol{u}\left( \boldsymbol{r},t \right)$ at the location $\boldsymbol{r}$ is practically determined by the ensemble average over randomly oriented thermal forces and, therefore, $\left\langle\nabla\cdot\boldsymbol{\xi} \right\rangle=0$.

Next, we describe in detail the particular situation illustrated in Fig. 1 of the main text. We consider only external forces acting along the radial direction and the local flux $\boldsymbol{J}\left( \boldsymbol{r},t \right)$of particles through a generic cylindrical surface.

If the applied field is azimuthally symmetric, the problem can be fully described as a one-dimensional process. Such an external force acting locally can be easily generated electromagnetically, for instance, by a structured field such as an optical vortex beam^3^. Rigorously, the force field comprises both conservative and non-conservative components^3^, and the particles are subject to longitudinal and transversal forces^4^. We will first analyze the effect of the transversal forces, which are dominated by intensity gradients^5^.

For particles much smaller than the wavelength, the time averaging electromagnetic force can be formally written as the sum of three components $\boldsymbol{F}_{\boldsymbol{ext}}=\frac{1}{4}Re\left\{ \alpha\right\}\nabla I+\frac{\sigma}{c}\boldsymbol{S}+\frac{\sigma}{c}\left( \nabla\times\boldsymbol{L}_{\boldsymbol{s}} \right)$ that depend on intensity $I$*,* Poynting vector$\boldsymbol{S}$*,* and spin flux$\boldsymbol{L}_{\boldsymbol{s}}$ ^4^. Here the time averaging is over time scales that are much larger than the frequency of the electromagnetic waves. The force will also depend on scattering cross-section $\sigma$ of the particle and its polarizability $\alpha$. Here, we assumed that the scattering of the particle suspension is weak such that the beam profile could be presented analytically. When the colloidal system is subjected to a linearly polarized optical vortex field with topological charge $m$, beam waist $w,$ and a slowly varying envelope $A\left( t \right)$,

$U\left( r,\theta,z_{0};t \right)=A\left( t \right)e^{-\frac{r^{2}}{w^{2}\left( z_{0} \right)}}\frac{r^{m}e^{im\theta}}{w^{m}\left( z_{0} \right)}=A\left( t \right)U^{'}\left( r,\theta,z_{0} \right)$, (S3)

the applied external force can be written as

$\boldsymbol{F}_{ext}\left( \boldsymbol{r},t \right)=\frac{I}{4}Re\left\{ \alpha\right\}\left( -\frac{4r}{w^{2}}+\frac{2m}{r} \right)\hat{\boldsymbol{r}}+\sigma\varepsilon_{0}\omega cI\left( \frac{m}{r}\hat{\boldsymbol{\theta}}\mathbf{+}k\hat{\mathbf{z}} \right)$, (S4)

where $I=\left| U\left( r,\theta,z_{0};t \right) \right|^{2}.$ In these conditions, the instantaneous velocity of a typical particle becomes

$\boldsymbol{v}\left( \boldsymbol{r},t \right)=\frac{I}{4\gamma}Re\left\{ \alpha\right\}\left( -\frac{4r}{w^{2}}+\frac{2m}{r} \right)\hat{\boldsymbol{r}}+I\frac{\sigma\varepsilon_{0}\omega c}{\gamma}\left( \frac{m}{r}\hat{\boldsymbol{\theta}}\mathbf{+}k\hat{\mathbf{z}} \right)+\frac{\boldsymbol{\xi}\left( t \right)}{\gamma}$, (S5)

where the first two terms represent the contributions of the conservative and nonconservative external forces.

To estimate the effect on the local density of particles, one has to evaluate the gradient of the local velocity field $\boldsymbol{u}\left( \boldsymbol{r},t \right)$ in Eq. (S2). At a generic point $r$, the radial gradient is

$\nabla\cdot\boldsymbol{u}\left( r,t \right) =\frac{I}{r}Re\left\{ \alpha\right\}\left[ -\frac{4r^{3}}{w^{4}}-\frac{r}{w^{2}}\left( 4m+2 \right)+\frac{m^{2}}{r} \right]$, (S6)

which means that the conservative part of external force is responsible for a radial flux of particles through the surface of the virtual cylinder in Fig. 1 (See main text). As a result, assuming that the external field is applied at time 0, over a period of time $t$, the colloidal density varies like

$\rho\left( r,t \right)={\rho_{0}e}^{-f\left( r \right)\int_{0}^{t} A\left( t^{'} \right)dt^{'}}$, (S7)

with a rate of change $f\left( r \right)=\frac{dF(r,t)}{dt}=\frac{1}{r}\frac{dI}{dt}Re\left\{ \alpha\right\}\left[ -\frac{4r^{3}}{w^{4}}-\frac{r}{w^{2}}\left( 4m+2 \right)+\frac{m^{2}}{r} \right].$ If the field amplitude is constant during the interval $\Delta t$, a steady state establishes where the colloidal density acquires a radial profile $\rho\left( r,t \right)$ $={\rho_{0}e}^{-f\left( r \right)t}$determined by the properties of the optical field. As long as the field is applied, the density at the radial position$r$ decreases exponentially with a rate determined by 1/$f\left( r \right).$ Over the cross-section of the entire virtual cylinder of radius $R$, the total density will vary as $\Upsilon\left( t \right)=\int_{0}^{R} \rho\left( r,t \right)rdr.$ The level of this density variation is controlled by the structure and the intensity of the applied field. We have investigated both the effect of intensity and the topological charge of the beam in the next two sections, respectively.

Once the external force field is turned off, say at time $t=t_{0},$ the particles return to their state of free thermal motion with a velocity$\boldsymbol{v}\left( t \right)=\frac{k_{b}T}{\gamma}\boldsymbol{\xi}\left( t \right)$. Thus, at time $t=t_{0}+\Delta t,$ the displacement of an arbitrary particle located initially at $\boldsymbol{r}_{0}$ will be $\boldsymbol{r}-\boldsymbol{r}_{0}=\frac{k_{b}T}{\gamma}\int_{t_{0}}^{t_{0}+\Delta t} \boldsymbol{\xi}\left( t \right)dt.$ One can easily find that $\rho\left( r,t \right)=\frac{\rho\left( r_{0},t_{0} \right)}{\sqrt{2\pi B}}e^{\frac{{(r-A)}^{2}}{2B}}$ in terms of the mean position $A=\left\langle r(t) \right\rangle=r_{0}$ and mean square displacement $B=\left\langle{(r-A)}^{2} \right\rangle=D(t-t_{0}).$ Thus, at a radial position$r_{0}$, the density will evolve in time as

$\rho\left( r,t \right)=\frac{\rho\left( r_{0},t_{0} \right)}{\sqrt{2\pi D(t-t_{0})}}e^{\frac{{(r-r_{0})}^{2}}{4D(t-t_{0})}}$ (S8)

at a rate determined solely by the thermal diffusion coefficient $D$.

# Effect of radial field intensity

In this section we evaluate the effect of field magnitude on the density variation based on the model described in the main text, Eq. (2) (as well as Eq. (S7)), within a virtual cylinder of diameter 1.2mm. As expected, the particle density decreases more rapidly when higher power is applied, which leads to a decrease of the overall optical density within the virtual cylinder.


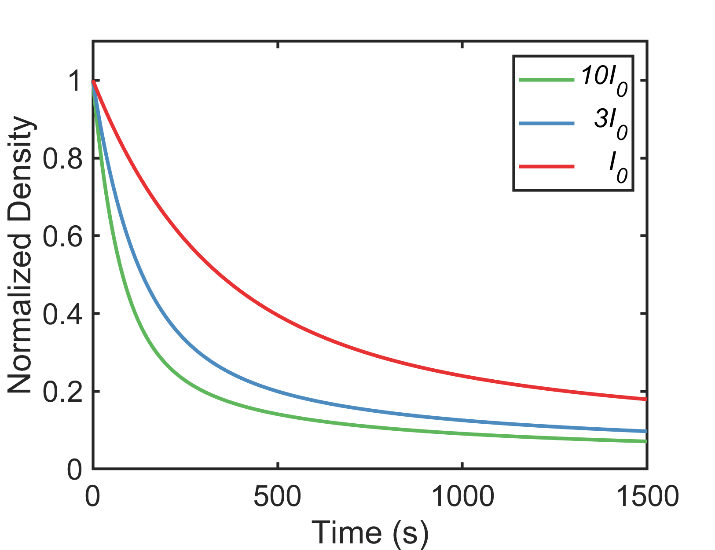


The density decrease determines an increase in the transmittance of a co-axial probe beam as shown here for a co-axial Gaussian beam of 0.5mm. The transmittance is evaluated according to the Lambert-Beer attenuation law. The optical density variations are calculated for 22 nm particles in water at λ=543nm. This dependence was verified experimentally as shown in Fig 4(b) in the main text.


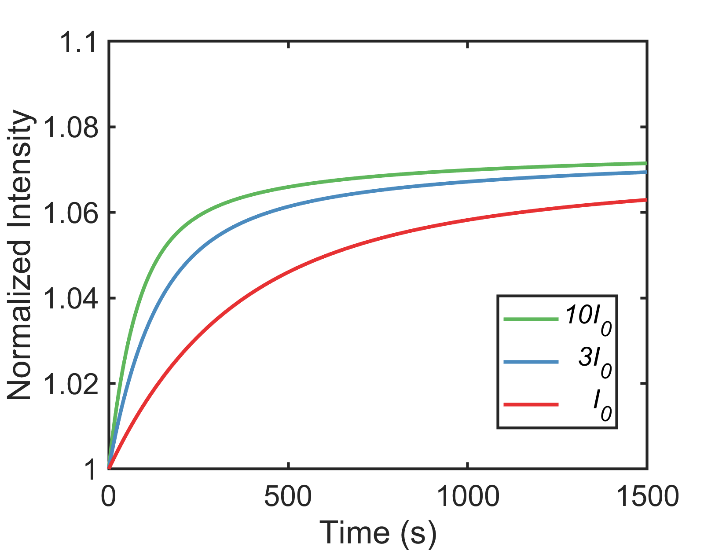


# Effect of topological charge of applied field

In this section we investigate the effect of topological charge of the vortex beam on the density variation based on the model described in the main text, Eq. (2) (as well as Eq. (S7)), within a virtual cylinder of diameter 1.2mm. As can be seen, the particle density within the cylinder decreases faster with smaller topological charge, which leads to a decrease of the optical density within the cylinder.


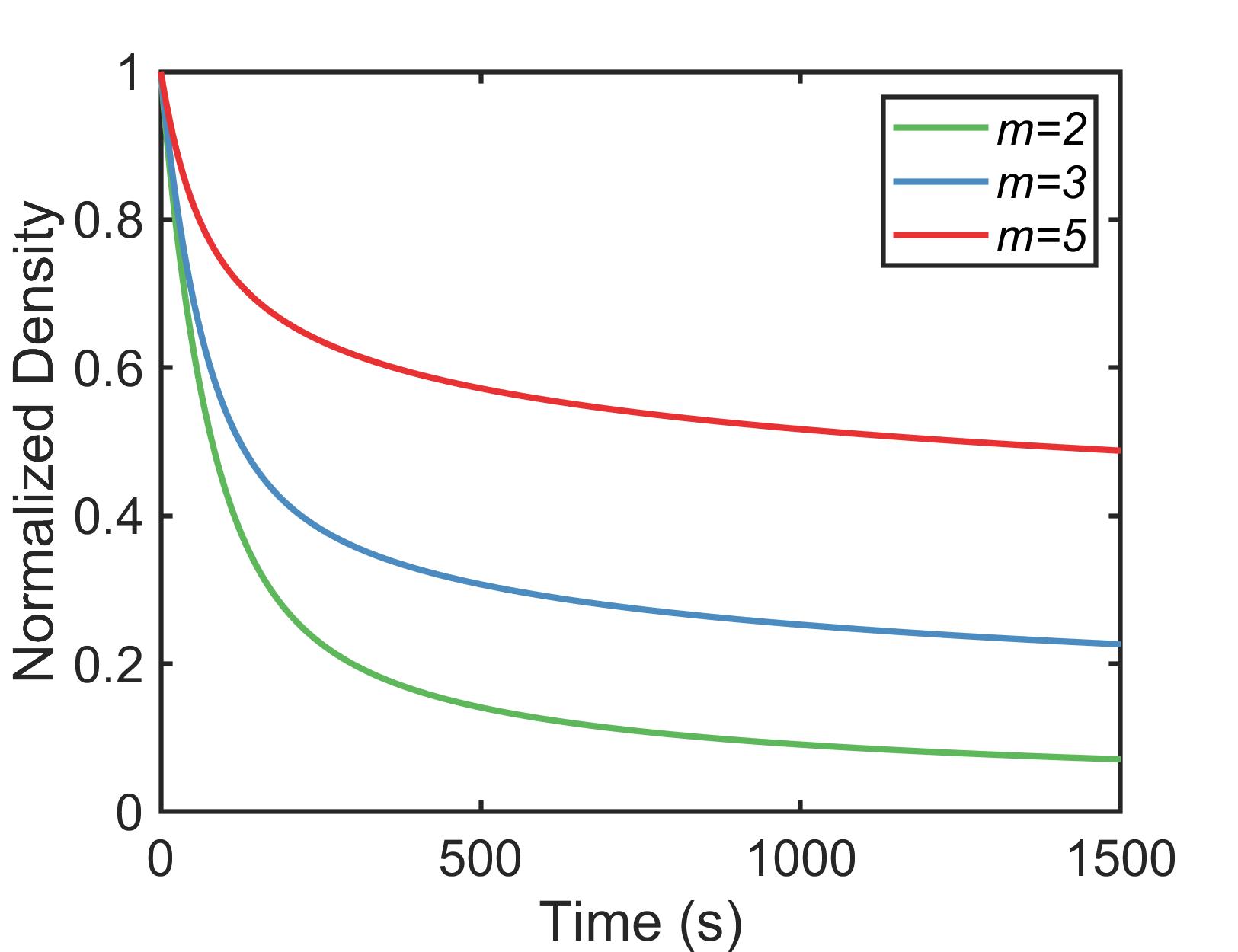


The density decrease determines an increase in the transmittance of a co-axial probe beam as shown here for a co-axial Gaussian beam of 0.5mm. The transmittance is evaluated according to the Lambert-Beer attenuation law. The optical density variations are calculated for 22 nm particles in water at λ=543nm.


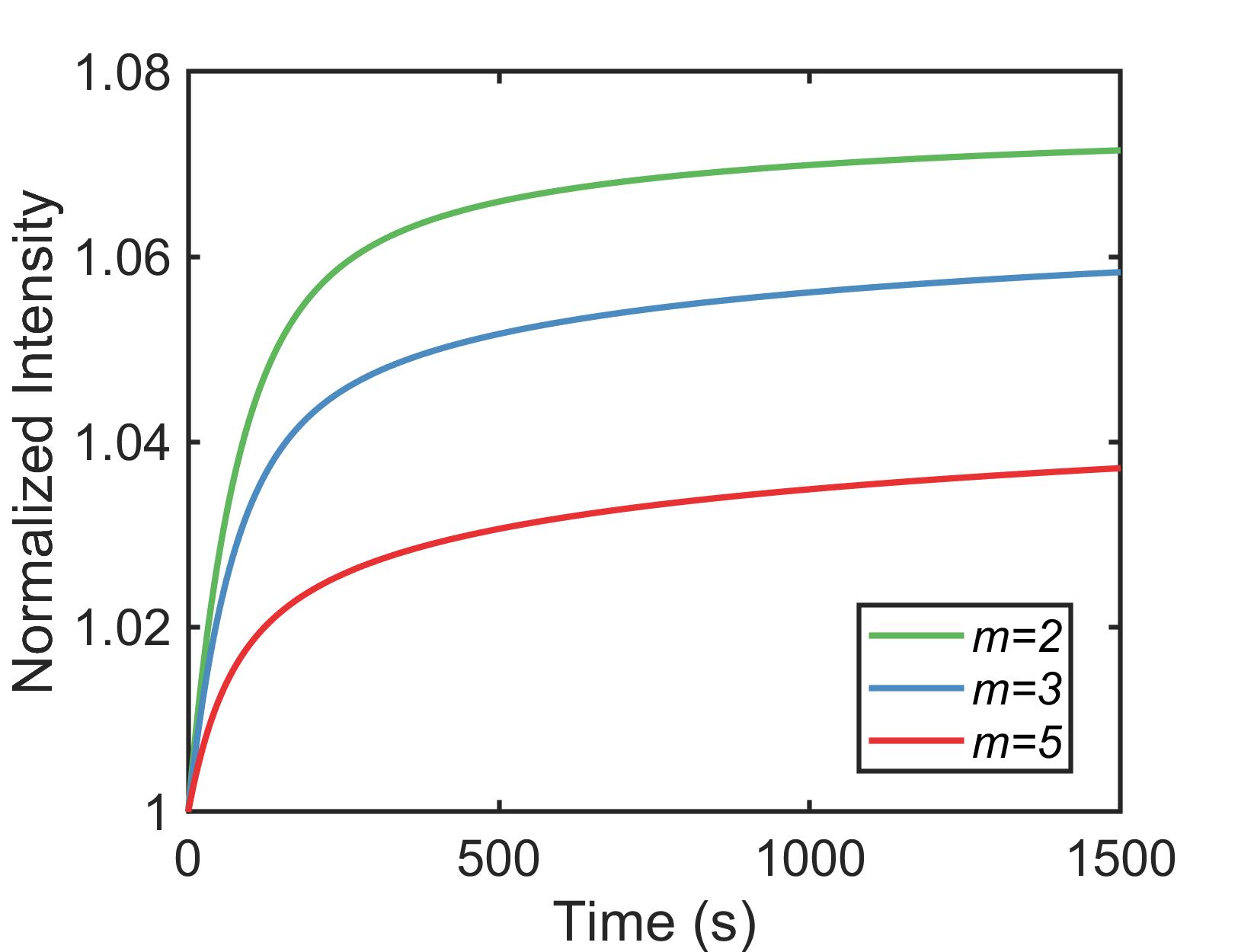


# Density variation for different types of particles

Experimental results for 3μm polystyrene particles in DI water are summarized in Fig. S1. The results for the 22nm particle colloidal with the same concentration are also included for comparison. Similar to the experiments described in the main text, the green control beam is turned on only between 150 and 1350 seconds during the experiment (Fig. S1(a)).

Similar trends are observed for the evolution of longitudinal particle density (Fig. S1(b)). In this case, however, one cannot find an analytical description of the velocity fields like in Eq. (S6) (see section 1) as the complex electromagnetic interaction can only be described numerically. On the other hand, as shown in Fig. S1(c), the decrease of the increment of intensity will both follow the trends described in the main text. As a pure Fick diffusion process, its rate of decrease (slope in Fig. S1(c)) will be inversely proportional to the hydrodynamic diameter of the particle.

**Figure S1**: Experimental results on particles at different scale. (a) The normalized transmittance for different particle size. Note that the green vortex beam is on between 150-1350s (between two dashed lines). (b) The regime right after the green beam is turned on. (c) The regime after the green control beam is turned off.


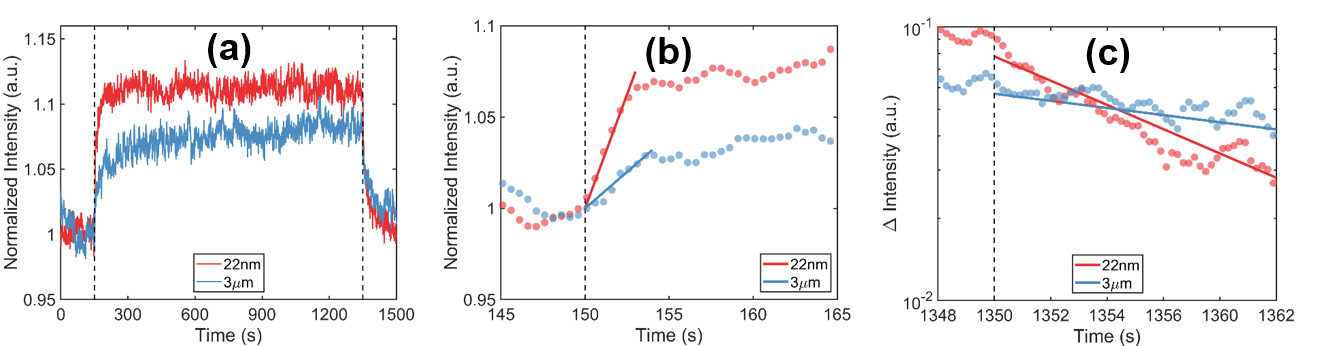


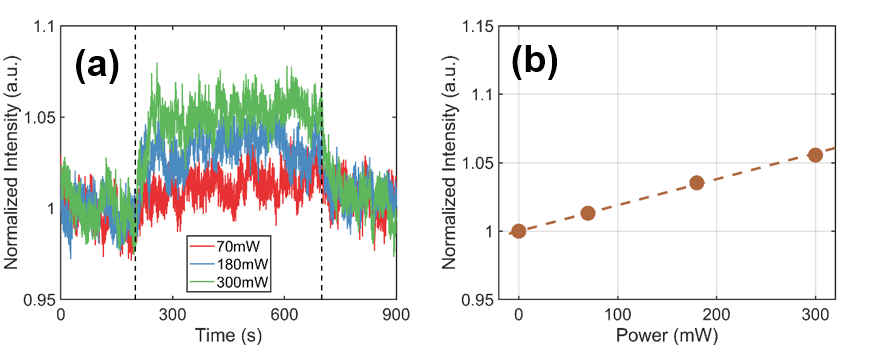
The magnitude of the transmitted probe intensity increases with the laser power in a manner similar to the one presented in the main text for the 22nm colloidal system. The maximum intensity varies linearly with the power of the external vortex field as described in the main text. Note that the effects of radiation intensity and particle size are independently of each other.

**Figure S2**: (a) Evolution of normalized transmittance for different power of the vortex beam (b) Normalized maximum intensity value as a function of the initial power in the vortex beam.

# References

1. Ross, S., Morrison, I. D. & Ross, I. *Colloidal Systems and Interfaces*. (Wiley, 1988).

2. RATHAKRISHNAN, R. *Fluid Mechanics: An Introduction*. (PHI Learning Pvt. Ltd., 2012).

3. Sukhov, S. & Dogariu, A. Non-conservative optical forces. *Rep. Prog. Phys.* **80**, 112001 (2017).

4. Albaladejo, S., Marqués, M. I., Laroche, M. & Sáenz, J. J. Scattering forces from the curl of the spin angular momentum of a Light field. *Phys. Rev. Lett.* **102**, 113602 (2009).

5. Ashkin, A. Optical trapping and manipulation of neutral particles using lasers. *PNAS* **94**, 4853–4860 (1997).
